# Ascertaining the biochemical function of an essential pectin methylesterase in the gut microbe *Bacteroides thetaiotaomicron*

**DOI:** 10.1074/jbc.RA120.014974

**Published:** 2021-01-13

**Authors:** Cheng-Jie Duan, Arnaud Baslé, Marcelo Visona Liberato, Joseph Gray, Sergey A. Nepogodiev, Robert A. Field, Nathalie Juge, Didier Ndeh

**Affiliations:** 1 State Key Laboratory for Conservation and Utilization of Subtropical Agro-bioresources, College of Life Science and Technology, Guangxi University, Nanning, Guangxi, China; 2 Institute for Cell and Molecular Biosciences, Newcastle University, Newcastle upon Tyne, United Kingdom; 3 Laboratório Nacional de Ciência e Tecnologia do Bioetanol, Centro Nacional de Pesquisa em Energia e Materiais, Campinas, Brazil; 4 Department of Biological Chemistry, John Innes Centre, Norwich, United Kingdom; 5 Department of Chemistry and Manchester Institute of Biotechnology, University of Manchester, Manchester, UK; 6 Quadram Institute Bioscience, Norwich, United Kingdom

**Keywords:** enzyme structure, glycobiology, microbiome, microbiology, pectin methylesterase, Bacteroides thetaiotaomicron, enzyme, BT1017, rhamnogalacturonan II

## Abstract

Pectins are a major dietary nutrient source for the human gut microbiota. The prominent gut microbe *Bacteroides thetaiotaomicron* was recently shown to encode the founding member (BT1017) of a new family of pectin methylesterases essential for the metabolism of the complex pectin rhamnogalacturonan-II (RG-II). However, biochemical and structural knowledge of this family is lacking. Here, we showed that BT1017 is critical for the metabolism of an RG-II–derived oligosaccharide ΔBT1017oligoB generated by a BT1017 deletion mutant (ΔBT1017) during growth on carbohydrate extract from apple juice. Structural analyses of ΔBT1017oligoB using a combination of enzymatic, mass spectrometric, and NMR approaches revealed that it is a bimethylated nonaoligosaccharide (GlcA-β1,4-(2-*O*-Me-Xyl-α1,3)-Fuc-α1,4-(GalA-β1,3)-Rha-α1,3-Api-β1,2-(Ara*f*-α1,3)-(GalA-α1,4)-GalA) containing components of the RG-II backbone and its side chains. We showed that the catalytic module of BT1017 adopts an α/β-hydrolase fold, consisting of a central twisted 10-stranded β-sheet sandwiched by several α-helices. This constitutes a new fold for pectin methylesterases, which are predominantly right-handed β-helical proteins. Bioinformatic analyses revealed that the family is dominated by sequences from prominent genera of the human gut microbiota, including *Bacteroides* and *Prevotella*. Our re-sults not only highlight the critical role played by this family of enzymes in pectin metabolism but also provide new insights into the molecular basis of the adaptation of *B. thetaiotaomicron* to the human gut.

The human large intestine is home to a large microbial community termed the human gut microbiota (HGM), which has substantial impact on the health and physiology of its host. Pectins, which are a major component of plant-based diets, have been shown to exert a significant selective pressure on HGM species (1, 2, 3) and hence have great potential as tools to manipulate the HGM. Pectins are defined as d-galacturonic acid–containing plant cell wall polysaccharides. The pectin macrostructure consists of three major polysaccharides: rhamnogalacturonan-I (RG-I), rhamnogalacturonan-II (RG-II), and homogalacturonan (4). Of these, RG-II is the most complex, consisting of several heterogenous side chains (A, B, C, D, E, and F), which are linked to a backbone of d-galacturonic acid (GalA) residues (Fig. 1*A*) (5). In total, RG-II contains at least 22 distinct glycosidic linkages and 13 different monosaccharides (Fig. 1*A*). The structure of RG-II is highly conserved; however, there is some variation in RG-II between plant species particularly at the termini of side chain B and in the methylation pattern of side chain A, as described previously (6, 7).

**Figure 1:**
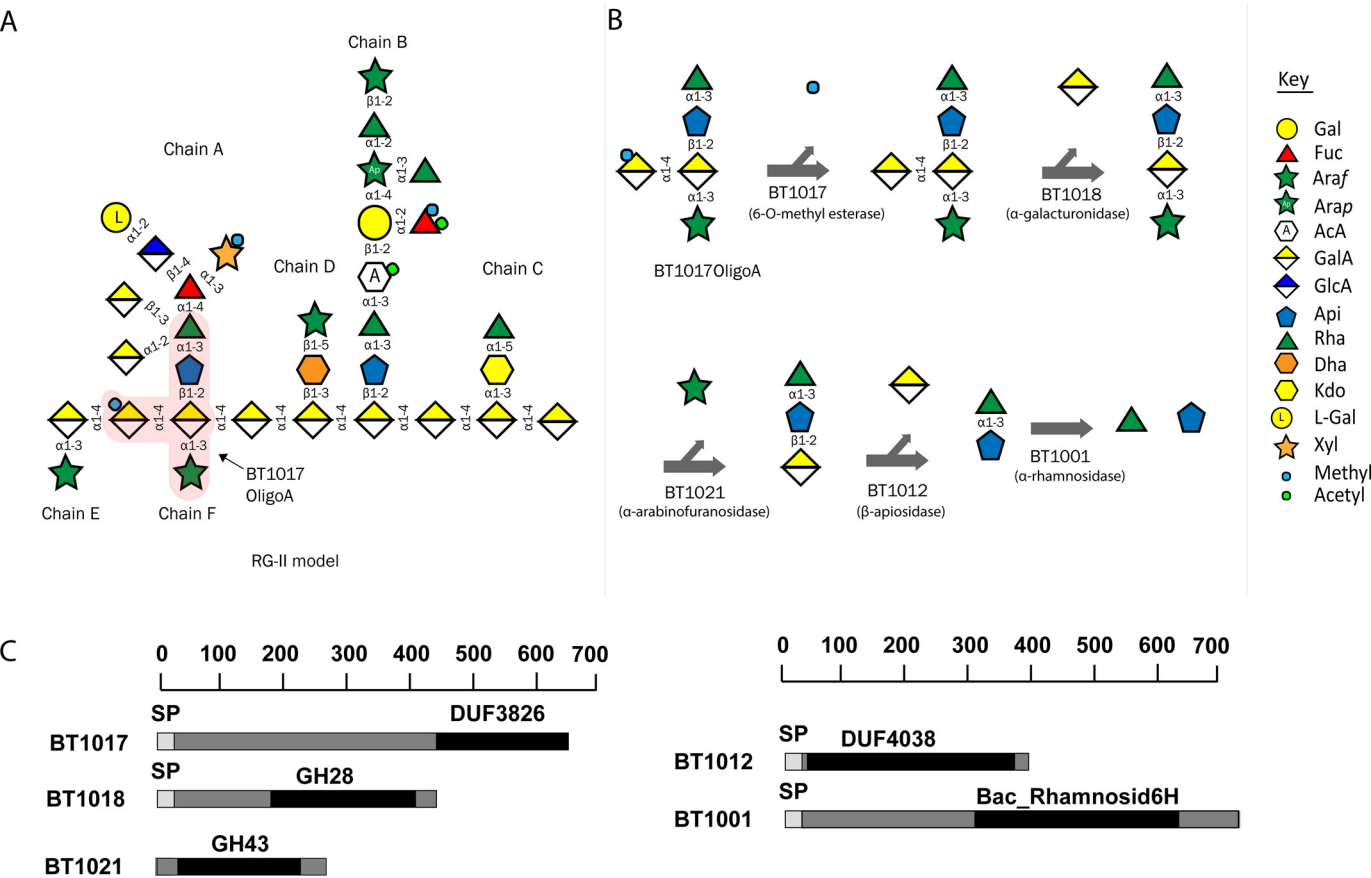
**Structure and degradation of pectin RG-II.***A*, structural model of RG-II showing various side chains and ΔBT1017oligoA (*pink shading*). *B*, pathway showing the five enzymes (A5) required for complete ΔBT1017oligoA degradation in the human gut microbe *B. thetaiotaomicron*. *Gal*, galactose; *Fuc*, fucose; *Araf*, arabinofuranose; *Arap*, arabinopyranose; *AcA*, aceric acid; *GalA*, galacturonic acid; *GlcA*, glucuronic acid; *Api*, apiose; *Rha*, rhamnose; *Dha*, 3-deoxy-d-lyxo-2-heptulosaric acid; *Kdo*, 3-deoxy-d-manno-2-octulosonic acid; *L-Gal*, l-galactose; *Xyl*, xylose; *Methyl*, methyl group; *Acetyl*, acetyl group. *C*, modular architecture of BT1017 and various proteins involved in ΔBT1017oligoA degradation.

*Bacteroides thetaiotaomicron* is a prominent member of the HGM, equipped with a large repertoire of carbohydrate-active enzymes (CAZymes) and considered as a generalist being able to forage on a wide range of dietary or host glycans (for a review see Ref. 4). *B. thetaiotaomicron* has the ability to cleave 21 of the 22 glycosidic linkages in RG-II (except that in the disaccharide 2-*O*-Me-Xyl-α1,3-Fuc) (5). Among the *B. thetaiotaomicron* repertoire, several founding members of novel CAZyme families were characterized including a pectin methylesterase (PME) BT1017. BT1017 was shown to remove the 6-*O*-methyl decoration of GalA in the homogalacturonan backbone of RG-II, therefore playing a critical role in enabling access to the rest of the RG-II structure by other RG-II–degrading enzymes (5). Currently more than 18 carbohydrate esterase families have been assigned, according to the CAZyme database (8); this topic was recently reviewed by Nakamura *et al.* (9). Of the 18 families, CE8 is the only family that contains PMEs. BT1017, however, displays no sequence similarity to CE8 esterases, and hence the structural basis for its catalytic function is unknown. When cultured in media containing extensively purified apple RG-II as a sole carbon source, a *B. thetaiotaomicron* genetic mutant lacking the BT1017 enzyme (ΔBT1017) produces a pentasaccharide Rha-α1,3-Api-β1,2-(Ara*f*-α1,3)-(6-*O*-Me-GalA-α1,4)-GalA here re-ferred to as ΔBT1017oligoA (Fig. 1*A*) (5). The complete degradation of ΔBT1017oligoA requires five enzymes (BT1017, BT1018, BT1021, BT1012, and BT1001) collectively referred to here as A5 (Fig. 1*B*). BT1017 cleaves the 6-*O*-methylester linkage from the backbone GalA; BT1018 (α-galacturonidase) cleaves the glycosidic linkage between the two backbone GalA residues; BT1021 (α-arabinofuranosidase) cleaves the linkage between Ara*f* (chain F) and the reducing end GalA; BT1012 (β-apiosidase) cleaves the linkage between Api and the reducing end GalA; and finally BT1001 (α-rhamnosidase) cleaves the linkage between Rha and Api in the Rha–Api disaccharide (Fig. 1*B*).

In the present study, we showed that the *B. thetaiotaomicron* mutant ΔBT1017, when cultured in carbohydrate extract from apple juice (CEAJ) as a sole carbon source, generates a second oligosaccharide (hereafter referred to as ΔBT1017oligoB). Our structural analyses revealed that ΔBT1017oligoB is a dimethylated nonasaccharide containing components of the RG-II backbone and its side chains. We characterized the kinetic properties of BT1017, showing that the enzyme has a low turnover against apple RG-II, ΔBT1017oligoA, and ΔBT1017oligoB and hence may represent a rate-limiting step during RG-II metabolism. We revealed that BT1017 is a serine esterase with an α/β-hydrolase fold and hence has not evolved from the progenitor protein that gave rise to the CE8 family of PMEs, which are predominantly comprised of right-handed β-helices.

## Results

### Characterization of ΔBT1017oligoB

**Figure 2:**
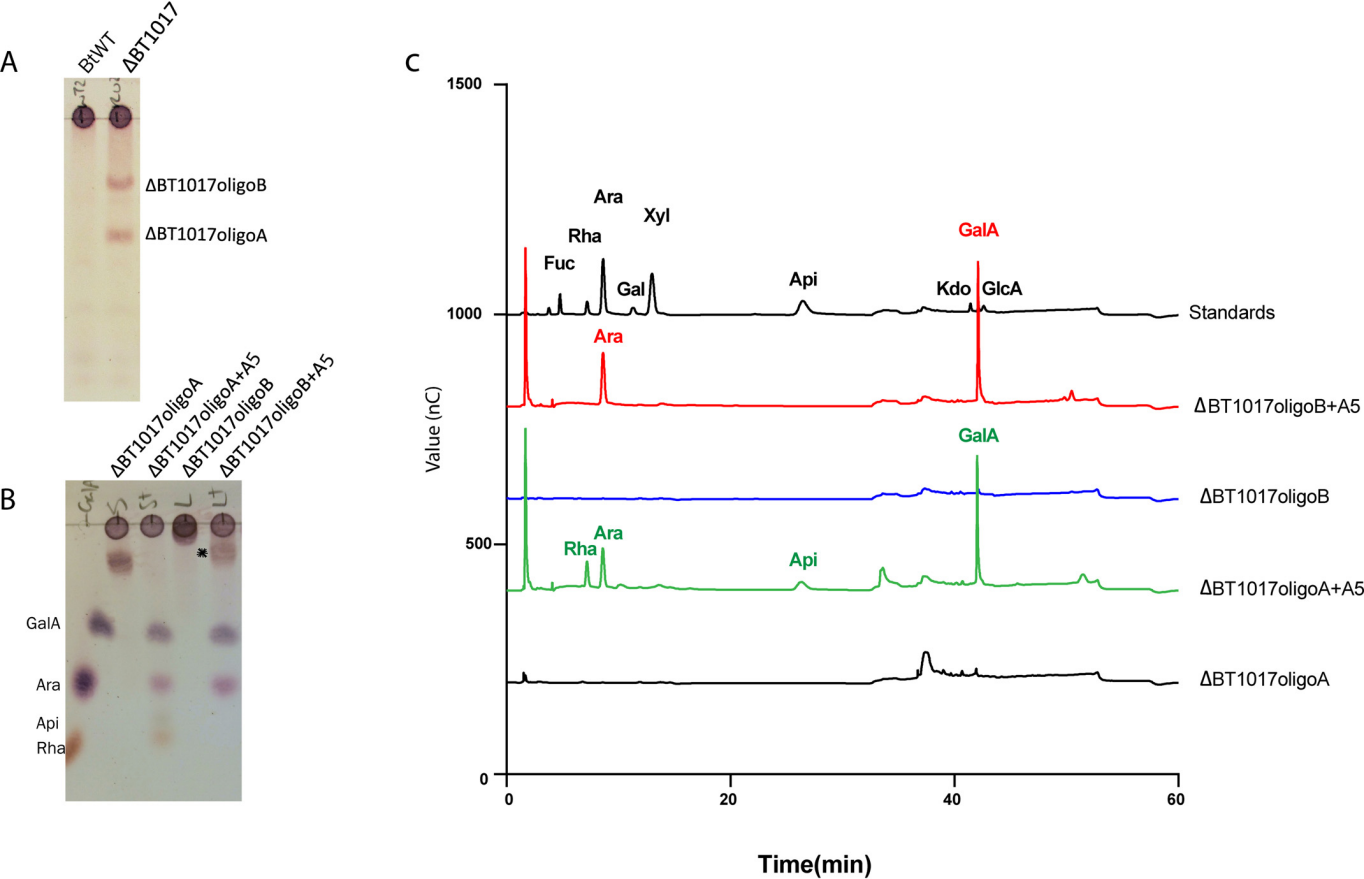
***B. thetaiotaomicron* deletion mutant ΔBT1017 generates two oligosaccharides, ΔBT1017oligoA and ΔBT1017oligoB, during growth on CEAJ.***A*, TLC analyses of culture supernatants from *B. thetaiotaomicron* WT (*BtWT*) and ΔBT1017 cells post-growth on CEAJ. *B*, digestion of ΔBT1017oligoA and B with a mixture of ΔBT1017oligoA-degrading enzymes (A5) containing BT1017, BT1018, BT1021, BT1012, and BT1001. Each substrate was treated with A5, and the reaction was stopped and analyzed by TLC. The product marked with an *asterisk* is the leftover of ΔBT1017oligoB after digestion with the mixture of A5 enzymes. *C*, HPLC analyses of samples in ΔBT1017oligoB.

*B. thetaiotaomicron* ΔBT1017 deletion mutant was cultured on CEAJ for 48 h to stationary phase (*A*_600 nm_ ∼1.0), and TLC was first used to analyze the culture supernatants. The data showed that ΔBT1017 generates two oligosaccharides, defined as ΔBT1017oligoA and ΔBT1017oligoB (Fig. 2*A*). Both sugars were purified by size-exclusion chromatography and treated independently with a mixture of recombinant A5 enzymes including BT1017, BT1018 (α-galacturonidase), BT1021 (α-arabinofuranosidase), BT1012 (β-apiosidase), and BT1001 (α-rhamnosidase), which target specific glycosidic linkages in RG-II (5). Unless otherwise stated, all the recombinant RG-II–degrading enzymes mentioned in this text were the same constructs used by Ndeh *et al.* (5) and lack the N-terminal signal peptide (SP) regions (Fig. 1*C*). The products of the enzymatic treatment were then analyzed by TLC and HPLC (Fig. 2, *B* and *C*). Digestion of ΔBT1017oligoA yielded GalA, Ara*f*, Rha and Api indicating that the molecule is the methylated pentasaccharide (Rha-α1,3-Api-β1,2-(Ara-α1,3)(6-*O*-Me-GalA-α1,4)-GalA, which was used to demonstrate the site of action of the PME in Ndeh *et al.* (5). The digestion of ΔBT1017oligoB, on the other hand, was incomplete, yielding only two monosaccharides (Ara*f* and GalA) and a third product of unknown identity (Fig. 2, *B* and *C*). The release of GalA and Ara*f* from ΔBT1017oligoB by ΔBT1017oligoA-specific enzymes BT1018 (α-galacturonidase) and BT1021 (α-arabinofuranosidase) suggests that ΔBT1017oligoB contains the backbone GalA and the side-chain F Ara*f* sugars characteristic of ΔBT1017oligoA (Fig. 1, *A* and *B*).

**Figure 3:**
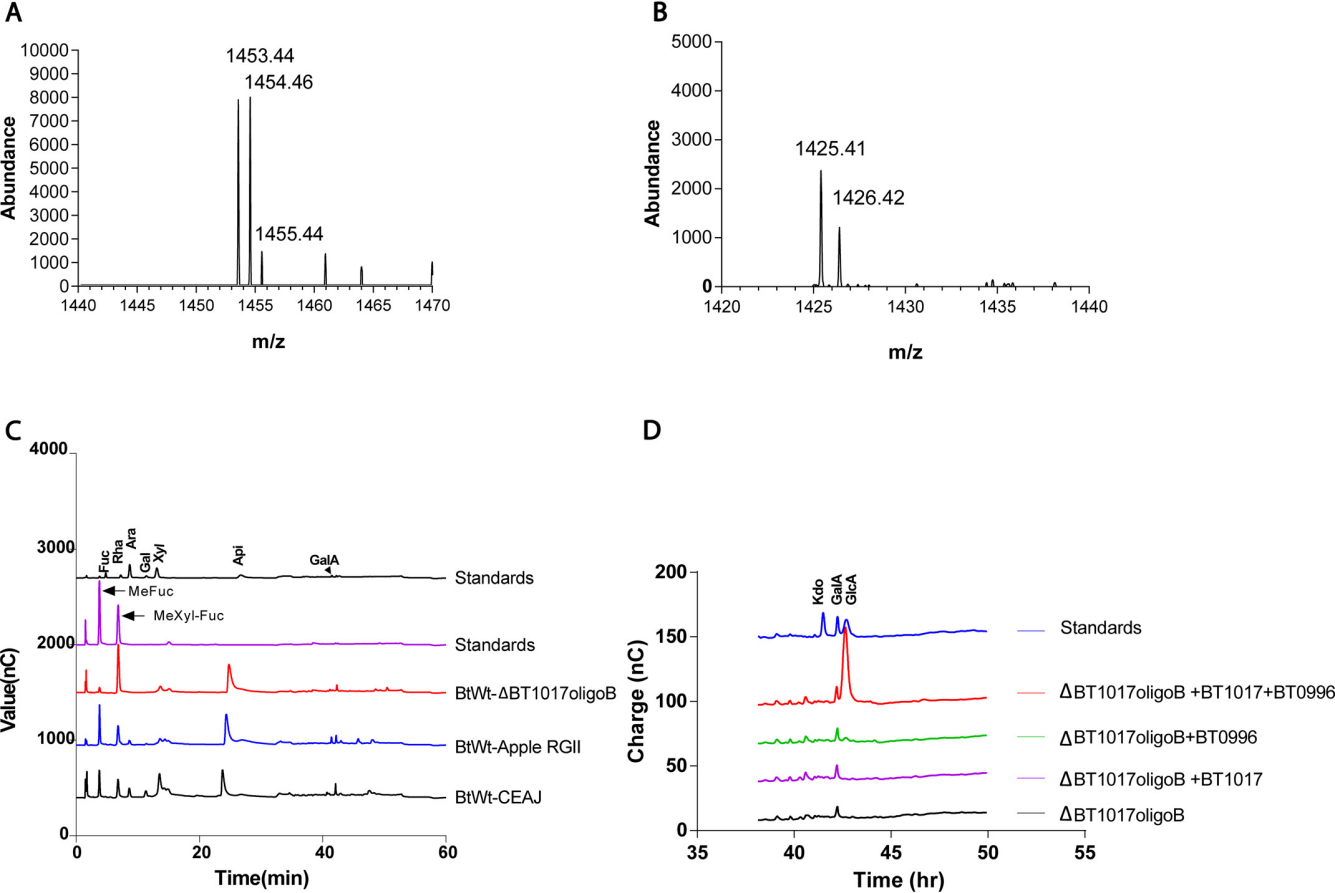
**Analyses of ΔBT1017oligoB by MS and HPLC.***A*, MS of purified ΔBT1017oligoB. Masses presented are for ΔBT1017oligoB and fragments plus hydrogen ions [H]^+^. *B*, MS of purified ΔBT1017oligoB after digestion with BT1017. *C*, HPLC analyses of spent media following growth of *B. thetaiotaomicron* ΔBT1017 on ΔBT1017oligoB, apple RG-II, and CEAJ MeFuc: 2-*O*-Me-Fuc, Mexyl-Fuc: 2-*O*-Me-Xyl-α1,3-Fuc. *D*, HPLC data showing activity of BT0996 and BT1017 on ΔBT1017oligoB.

To determine the full structure of ΔBT1017oligoB, a combination of MS, enzymatic and NMR analyses were performed. First, MS data revealed that ΔBT1017oligoB has a protonated molecular mass ([M + H]^+^) of 1453.44 Da (Fig. 3*A*). When treated with BT1017, the mass of ΔBT1017oligoB decreased by 28.03 Da (Fig. 3*B*), corresponding to the loss of two methyl groups. This suggests that ΔBT1017oligoB contains two ester-linked methyl groups that were hydrolyzed by the BT1017 PME. Second, when WT *B. thetaiotaomicron* was cultured on ΔBT1017oligoB, the bacterium accumulated the disaccharide 2-*O*-Me-Xyl-α1,3-Fuc, which is unique to side chain A of RG-II but not present in ΔBT1017oligoA (Fig. 3*C*). The sugar Api was also detected. These results demonstrate that ΔBT1017oligoB contains components of ΔBT1017oligoA and additional sugars from RG-II side chain A. Last, ΔBT1017oligoB was shown to be susceptible to attack by the β-d-glucuronidase enzyme BT0996, which released GlcA (Fig. 3*D*). Because this required pretreatment with BT1017, this result suggests that at least one of the methyl decorations in ΔBT1017oligoB sterically hinders the activity of BT0996. Release of free GlcA is also an indication that ΔBT1017oligoB lacks the terminal l-Gal residue, which is α1,2-linked to GlcA at the nonreducing end of chain A (Fig. 1*A*).

Based on the above features of ΔBT1017oligoB (protonated mass of 1452.44 Da, presence of 2-*O*-Me-Xyl-α1,3-Fuc, GalA, Ara, Api, and GlcA (highlighted in Fig. S1A) and the absence of terminal l-Gal), two possible structures of de–methyl-esterified BT1017oligoB (ΔBT1017oligoB-2Me) were deduced from the known structure of RG-II (Fig. S1, B and C). These include ΔBT1017oligoB-2Me-α (GlcA-β1,4-(2-*O*-Me-Xyl-α1,3)-Fuc-α1,4-(GalA-α1,2)-Rha-α1,3-Api-β1,2-(Ara*f*-α1,3)-(GalA-α1,4)-GalA) and ΔBT1017oligoB-2Me-β (GlcA-β1,4-(2-*O*-Me-Xyl-α1,3)-Fuc-α1,4-(GalA-β1,3)-Rha-α1,3-Api-β1,2-(Ara*f*-α1,3)-(GalA-α1,4)-GalA).

**Figure 4:**
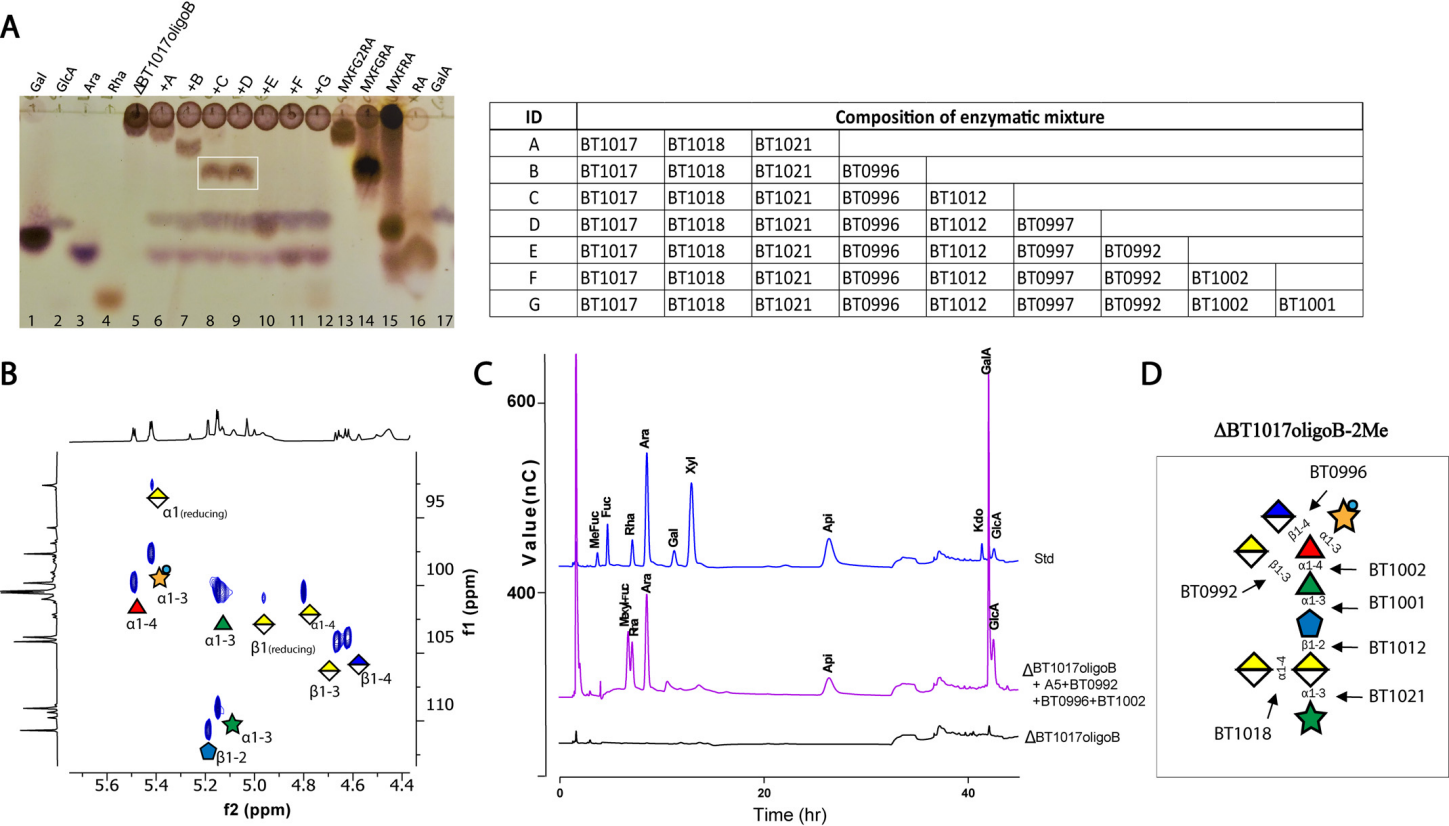
**Structural characterization of ΔBT1017oligoB.***A*, TLC analysis ΔBT1017oligoB after digestion with diverse combinations of RG-II–degrading enzymes. The *bands* in the *white rectangle* correspond to MXFGRA-x later confirmed to be MXFGRA-β (2-*O*-Me-Xyl-α1,3-Fuc-α1,4-(GalA-β1,3)-Rha-α1,3-Api). Other complex sugar standards include MXFG2RA (2-*O*-Me-Xyl-α1,3-Fuc-α1,4-(GalA-α1,2)(GalA-β1,3)-Rha-α1,3-Api), MXFRA (2-*O*-Me-Xyl-α1,3-Fuc-α1,4-Rha-α1,3-Api), and RA (Rha-α1,3-Api). *B*, HSQC NMR of anomeric region of ΔBT1017oligoB. *C*, HPLC analyses of ΔBT1017oligoB after complete hydrolysis with a mixture of ΔBT1017oligoB-degrading enzymes. *D*, model of ΔBT1017oligoB-2Me showing the cleavage sites of various ΔBT1017oligoB-degrading enzymes.

Both sugars differ by the presence of either α1,2- or β1,3-linked GalA (underlined). To determine which of them corresponded to ΔBT1017oligoB-2Me, enzymes targeting all linkages in the predicted sugars (ΔBT1017oligoB-2Me-α and ΔBT1017oligoB-2Me-β) were used to sequentially digest ΔBT1017oligoB. The first set of recombinant enzymes collectively referred to here as B5 enzymes include BT1017, BT1018, BT1021, BT0996, and BT1012. These together should cleave the two ester groups, the backbone α1,3-linked GalA, the side chain F α1,3-linked Ara*f*, the side chain A β1,4-linked GlcA, and the reducing end/backbone GalA residue, respectively (Fig. S1, D and E), to generate two possible pentasaccharide structures: MXFGRA-α (2-*O-*Me-Xyl-α1,3-Fuc-α1,4-(GalA-α1,2)-Rha-α1,3-Api) and MXFGRA-β (2-*O*-Me-Xyl-α1,3-Fuc-α1,4-(GalA-β1,3)-Rha-α1,3-Api), differing by the presence or absence of either α1,2- or β1,3-linked GalA (underlined). Digestion of ΔBT1017oligoB-2Me with a mixture of recombinant B5 enzymes (BT1017, BT1018, BT1021, BT0996, and BT1012) and subsequent analyses by TLC revealed the generation of a product that migrates to a similar extent as the sugar standard MXFGRA-β (Fig. 4*A*, *lane 8* in *white rectangle*). However, it was also possible that the product corresponded to MXFGRA-α because of the significant structural similarity to MXFGRA-β. As a result, it was referred to as MXFGRA-x. Both α1,2- and β1,3-GalA linkages in MXFGRA-α and MXFGRA-β have been shown to be specifically targeted by the enzymes BT0997 (α-galacturonidase) and BT0992 (β-galacturonidase), respectively (5) (Fig. S1, D and E); hence to determine whether the product contained α- or β-linked GalA residues, each of these enzymes (BT0992 and BT0997) was used to further digest MXFGRA-x. TLC analyses of the reaction showed that MXFGRA-x was digested by BT0992 but not by BT0997 (Fig. 4*A*, *lanes 9* and *10*, respectively), indicating that the exposed GalA residue in the product was β1,3-linked to Rha and that the pentasaccharide was MXFGRA-β. This was also confirmed by 2D HSQC NMR analyses of ΔBT1017oligoB, which detected ^1^H and ^13^C HSQC anomeric signals (δ_H_ 4.67 and δ_C_ 104.1) of β-GalA. The NMR analyses also revealed H1/C1 signals of all other carbohydrate residues in the anomeric region of the spectrum. These include signals for GalA-α1-4, Ara*f*-α1-3, Api-β1-2, Rha-α1-3, Fuc-α1-4, 2-*O*-Me-Xyl-α1-3, and GlcA-β1,4 (Fig. 4*B*), which were assigned by comparison with published data (10). Two weaker cross-peaks could be assigned to the anomeric center of the reducing-end GalA residue in the backbone of ΔBT1017oligoB. The full monosaccharide composition of ΔBT1017oligoB-2Me was confirmed by treatment of the sugar with a combination of A5 enzymes together with BT0996, BT0992, and other RG-II–degrading enzymes BT1002 (α-l-fucosidase) and BT1001 (α-l-rhamnosidase) and analyses of the digested sample by HPLC. The results showed that the enzymes degraded the sugar into all its constituent monosaccharides GlcA, GalA Rha Api Ara*f*, and the disaccharide 2-*O*-Me-Xyl-α1,3-Fuc (Fig. 4*C*). A model showing the cleavage sites of various enzymes on ΔBT1017oligoB is shown in Fig. 4*D*.

### Activity of BT1017 and kinetic parameters

**Figure 5:**
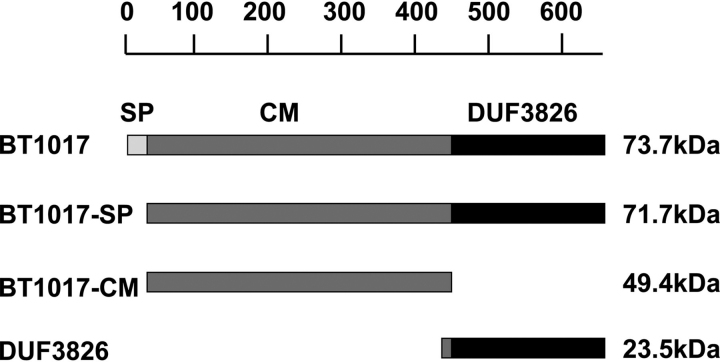
**Modular architecture of BT1017 showing various fragments expressed and tested for esterase and sugar-binding activity.** The models are drawn to scale. The *scale bar* above shows the amino acid sequence that was expressed.

Full-length BT1017 (BT1017-FL) is a 73.7-kDa protein consisting of a SP (positions 1–20), a sequence of unknown function (positions 20–400), or central module (CM) and a domain of unknown function DUF3826 (positions 400–600) (Figure 1, Figure 2, Figure 3, Figure 4, Figure 5). To determine the site of the esterase activity in BT1017, various recombinant fragments of the protein namely BT1017-SP (71.7 kDa; lacking the signal peptide), BT1017-CM (49.4 kDa; corresponding to CM), and BT1017-DUF3826 (23.5 kDa; corresponding to the DUF3826 domain) (Fig. 5) were expressed and tested against apple RG-II using a coupled spectrophotometric enzyme assay that measures released methanol (as described earlier (11)). Only BT1017-SP and BT1017-CM showed activity against the substrate (Table 1), indicating that BT1017-CM comprises the catalytic site. The optimal temperature and pH for BT1017 activity were determined to be 37 °C and 8.5, respectively (Fig. S2). To further explore the specificity of the enzyme, BT1017-SP was tested against various methyl- and acetyl-esterified substrates. The enzyme was active against ΔBT1017oligoA, ΔBT1017oligoB, 6-*O-*methyl galacturonic (Me-GalA), and 6-*O-*methyl glucuronic acid (Me-GlcA) but not methylpropionate, methylbutyrate, ethylpropionate ethylbutyrate, acetylated potato RG-I, and 4-nitrophenyl-acetate (Table 1). BT1017 thus appears to have a preference for methylated hexose sugars. The *K_m_* and *k*_cat_ of BT1017-SP and BT1017-CM against various substrates are reported in Table 1, showing that BT1017-SP has an ∼2-fold higher catalytic efficiency compared with BT1017-CM against more complex substrates such as apple pectin. BT1017-SP also showed a higher *k*_cat_/*K_m_* toward Me-GalA and ΔBT1017oligoB compared with Me-GlcA and ΔBT1017oligoB; however, the difference was less than 10-fold.

**Table 1:** Activity of BT1017-derived proteins and kinetic parameters NT, not tested; —, no activity; Me*, concentration of methyl groups in the substrate was used for the calculation of kinetic parameters.

Substrates	Parameters	BT1017 constructs			BT1017-CM mutants		
		BT1017-SP	BT1017-CM	BT1017-DUF3826	D352A	S282A	H388A
Apple RG-II^Me^*	*K_m_* (mm)	0.07 ± 0.01	0.1 ± 0.02	—	NT		
	*k*_cat_ (s^−1^)	0.4 ± 0.04	0.6 ± 0.06				
	*k*_cat_/*K_m_* (s^−1^ mm^−1^)	6 ± 1	5 ± 0.8				
Apple Pectin^Me^*	*K_m_* (mm)	0.04 ± 0.01	0.1 ± 0.004	—			
	*k*_cat_ (s^−1^)	0.3 ± 0.03	0.4 ± 0.06				
	*k*_cat_/*K_m_* (s^−1^ mm^−1^)	7 ± 2	3 ± 0.5				
BT1017OligoB	*K_m_* (mm)	0.2 ± 0.03	0.4 ± 0.07	NT	—	—	—
	*k*_cat_ (s^−1^)	0.2 ± 0.01	0.2 ± 0.01				
	*k*_cat_/*K_m_* (s^−1^ mm^−1^)	0.8 ± 0.1	0.5 ± 0.09				
BT1017OligoA	*K_m_* (mm)	3 ± 0.4	1 ± 0.2		—	—	—
	*k*_cat_ (s^−1^)	0.3 ± 0.02	0.2 ± 0.01				
	*k*_cat_/*K_m_* (s^−1^ mm^−1^)	0.1 ± 0.02	0.1 ± 0.02				
MeGalA	*K_m_* (mm)	0.3 ± 0.2	0.1 ± 0.02		—	—	—
	*k*_cat_ (s^−1^)	0.2 ± 0.01	0.1 ± 0.01				
	*k*_cat_/*K_m_* (s^−1^ mm^−1^)	0.9 ± 0.6	1 ± 0.2				
MeGlcA	*K_m_* (mm)	0.7 ± 0.1	0.5 ± 0.09				
	*k*_cat_ (s^−1^)	0.2 ± 0.001	0.1 ± 0.006				
	*k*_cat_/*K_m_* (s^−1^ mm^−1^)	0.2 ± 0.05	0.2 ± 0.04				

### 3D structural features of BT1017

**Figure 6:**
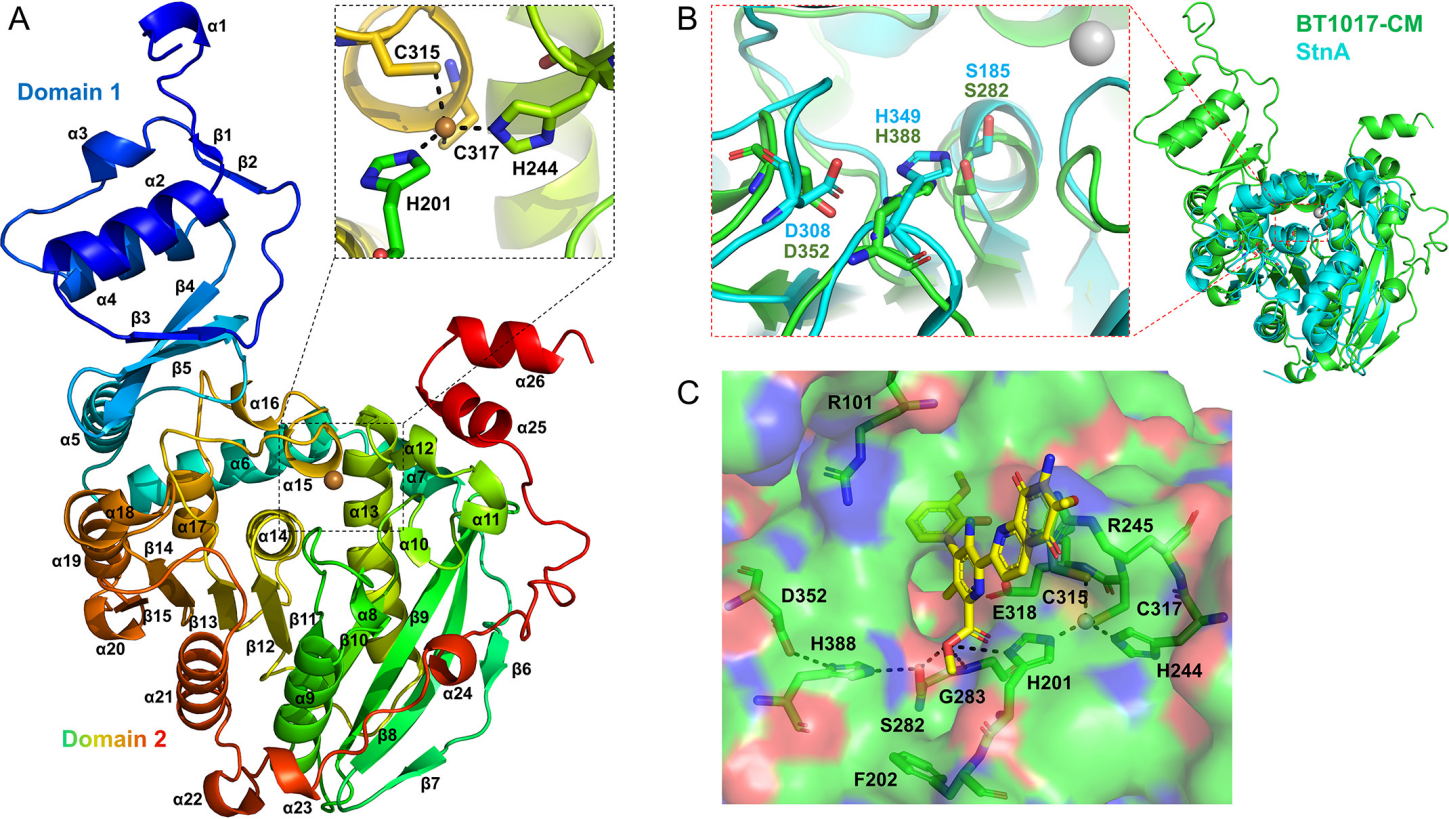
**3D structure of BT1017 (PDB entry**6GOC) **and superposition with streptonigrin methylesterase A (StnA).***A*, 3D cartoon structure of BT1017-CM shows two major domains (N-terminal domain 1 and C-terminal domain 2), composed of α/β folding. The model was colored as a spectrum from *blue* (N-terminal) to *red* (C-terminal). The top box illustrates a tetrahedral ion coordination system involving two cysteines and two histidines. *B*, structural alignment of BT1017-CM and StnA (16) showing the conserved catalytic residues Ser^282^, Asp^352^, and His^388^ (*left panel*). *C*, methylated ligand 10′-demethoxystreptonigrin (*yellow*) modeled from the superposition of BT1017-CM and StnA. A polar contact network connects the catalytic residues with substrate, the substrate with an oxyanion hole (composed of residues Sedr^282^, Gly^283^, and His^201^), and the zinc-binding site through His^201^. BT1017-CM surface is colored in *green* (apolar), *red* (polar-negative), and *blue* (polar-positive).

BT1017-SP could not be crystallized. BT1017-CM, however, generated crystals in the space group C2221 with one molecule in the asymmetric unit. The structure of the enzyme was determined by single-wavelength anomalous dispersion phasing and refined to 1.9 Å with an *R*_factor_ of 20.04% and an *R*_free_ of 24.09%. The final model (PDB entry 6GOC) of BT1017-CM consists of residues 20–462 of BT1017. The stereochemical quality of the model was assessed by validation tools in Coot and MolProbity (12, 13). The ratios of preferred and allowed regions from the Ramachandran plot are 96.61 and 3.17%, respectively. The statistics for data collection and refinement are summarized in Table 2. BT1017-CM comprises two major structural domains defined as domains 1 and 2 (Fig. 6*A*). Domain 1 forms what appears to be a flexible cap over the rest of the structure and consists of a globular α/β structure with one α-helical layer (α1–3) and one antiparallel β-strand layer (β1–3). Domain 2 displays a canonical α/β hydrolase fold in which a central twisted parallel and antiparallel 10-stranded β-sheet (β6–15) is sandwiched by several right-handed α-helices (Fig. 6*A*). The α/β-hydrolase fold is typical of esterases, lipases, and acetylases (9), consistent with the PME activity of BT1017. A C-terminal loop interspersed by five α-helices α22–26) likely represents a linker region to the DUF3826 domain (Fig. 6*A*). A single metal ion lies close to the potential active site of BT1017-CM (∼9Å from the catalytic serine) (Fig. 6, *A* and *B*). To determine its identity, the metal ion content of BT1017-CM was analyzed by inductively coupled plasma–MS. The highest amount of metal detected in the protein (∼10 µmol) was zinc (Zn^2+^ = 10.870 µmol), followed by calcium (Ca^2+^ = 1.929 µmol), cobalt (Co^2+^ = 1.141 µmol), and nickel (Ni^2+^ = 0.562 µmol), etc. (Table S1). The amount and stoichiometric concentration ratio of Zn^2+^ to BT1017-CM was approximately ∼1:1, suggesting that it is the metal ion close to the active site in BT1017-CM. The Zn^2+^ is shown to have a classical tetrahedral coordination geometry (14) potentially interacting with four ligands in its vicinity including Cys^315^, Cys^317^, His^201^, and His^244^ (Fig. 6*A*).

**Table 2:** Data statistics and refinement details of BT1017

**Data statistics**^a^	BT1017
Beamline	I24
Date	24/09/16
Wavelength (Å)	0.97889
Resolution (Å)	66.01–1.90 (1.94–1.90)
Space group	C 2 2 2_1_
Unit-cell parameters	
a (Å)	68.51
b (Å)	229.47
c (Å)	80.71
α = β = γ (°)	90
Unit-cell volume (Å^3^)	1,268,788
Solvent content (%)	42.5
No. of measured reflections	591,597 (30,870)
No. of independent reflections	50,611 (30,870)
Completeness (%)	100.0 (100.0)
Redundancy	11.7 (9.6)
CC_1/2_ (%)	0.998 (0.636)
/<σ(*I*)>	10.6 (2.2)
Anomalous completeness	99.8 (99.5)
Anomalous redundancy	5.8 (4.8)
**Refinement statistics**^a^	
*R*_work_ (%)	20.04
*R*_free_ (%)^b^	24.09
No. of non-hydrogen atoms	
No. of protein, atoms	3473
No. of solvent atoms	279
No. of Zn atoms	1
RMSD from ideal values	
Bond angle (°)	1.60
Bond length (Å)	0.013
Average B factor (Å^2^)	
Protein	35.1
Solvent	40.5
Zinc	30.8
Ramachandran statistics (Protein backbone)^c^	96.15/4.00/1.00


a The values in parentheses are for the highest resolution shell.

b 5% of the randomly selected reflections excluded from refinement.

c Calculated using MolProbity.

Structural similarity searches using PDBeFold (15) revealed that BT1017-CM is most similar to a putative xylan acetyl esterase BF1801 from *Bacteroides fragilis* (PDB code 3NUZ) with a *Q* score of 0.25 representing 245 Cα carbons overlapping with an RMSD of 1.85 Å and 20% sequence identity (Table S2). A superposition of both structures is shown in Fig. S3, showing significant alignment of several strands and helices of their α/β-hydrolase folds. The closest structural homolog with a verified methyl esterase activity is the enzyme streptonigrin methylesterase A (StnA) from a *Streptomyces* albus (16) with a Q score of 0.16, 195 Cα carbons overlapping with an RMSD of 2.45 Å and 11% sequence identity (Fig. 6*B*). StnA is an essential enzyme that de-esterifies intermediate products in the biosynthesis pathway of streptonigrin, an antitumor drug produced by *Streptomyces species* (16). Although the cores of both (StnA and BT1017-CM) 3D structures are very similar, parts of the loops surrounding the binding sites are more variable and no metal ion was present in BF1801 and StnA.

The C-terminal domain of BT1017 annotated as DUF3826 is similar to the proteins BT1022 (PDB code 3G6I; *E* value 4.8e^−5^) and BVU2916 (PDB code 3KDW; *E* value, 6.4e^−4^) of *B. thetaiotaomicron* and *B. vulgatus* respectively. Both are predicted to be carbohydrate-binding proteins. However, no binding to RG-II was observed for neither BT1017-DUF3826 nor BT1022 as shown by isothermal titration calorimetry (Fig. S4).

### Catalytic residues

BT1017-CM contains the Gly-X-Ser-X-Gly-Gly sequence motif (Fig. S5) characteristic of serine esterases and lipases (9), suggesting that the serine hydroxyl is the catalytic nucleophile in BT1017. Typically, the catalytic triad of serine esterases comprises an Asp, His and Ser. A proton relay system toward the aspartate decreases the p*K_a_* of the serine enabling it to function as the nucleophile. The histidine, in addition to participating in the proton relay, functions as the catalytic acid-base. The imidazole side chain protonates the alcohol leaving group following enzyme acetylation/methylation, and then activates a water molecule that then hydrolyzes the ester linkage between the enzyme and organic acid. Sequence alignment of BT1017-CM with selected functional and/or structurally similar serine carbohydrate esterases however only showed conservation of the catalytic Ser residue (Ser282) (Fig. S5) while the other residues of the triad (Asp352 and His388) were identified by structural alignment with StnA (16) (Fig. 6*B*) and confirmed experimentally by site-directed mutagenesis of each residue to alanine (S282A, D352A and H388A) (Table 1). The results showed that the location of the catalytic triad is conserved with Ser282 situated at the “nucleophile elbow” in a sharp turn extending from β12, with D352 and H388 positioned in loops emanating from β14 and β15, respectively. The overlay of a nucleophile mutant of StnA in complex with its substrate methyl 5-amino-6-(7-amino-6-methoxy-5,8-dioxo-5,8-dihydroquinolin-2-yl)-4-(2-hydroxy-3-methoxyphenyl)-3-methylpyridine-2-carboxylate (STM) provided further insight into the interaction of BT1017 with the methyl ester (Fig. 6*C*). The methyl group points into a shallow hydrophobic pocket in which the Phe202 forms its base. The carbonyl group of the carboxylate component of the ester bond fits into an oxyanion hole forming hydrogen bonds with the backbone nitrogen of Gly283 and Nδ1 of His201. These two polar contacts stabilize the negative charge of the carbonyl group that forms at the transition state. We were unable to obtain the crystal structure of BT1017-CM in complex with ligands, therefore preventing identification of the specificity determinants for GlcA or GalA. The oxyanion hole and hydrophobic pocket opens up onto a highly basic surface containing arginine residues (R101 and R245) that may form polar salt bridges of unmethylated GalA residues in the RG-II homogalacturonan backbone (Fig. 6*C*). There are no aromatic residues typical of sugar-binding sites and only a single polar residue, Glu318, in the vicinity of the region that is likely occupied by the uronic acid. The lack of significant polar interactions with the GlcA/GalA region of the substrate may explain the low sugar specificity displayed by BT1017.

### Phylogenetic analyses of BT1017

**Figure 7:**
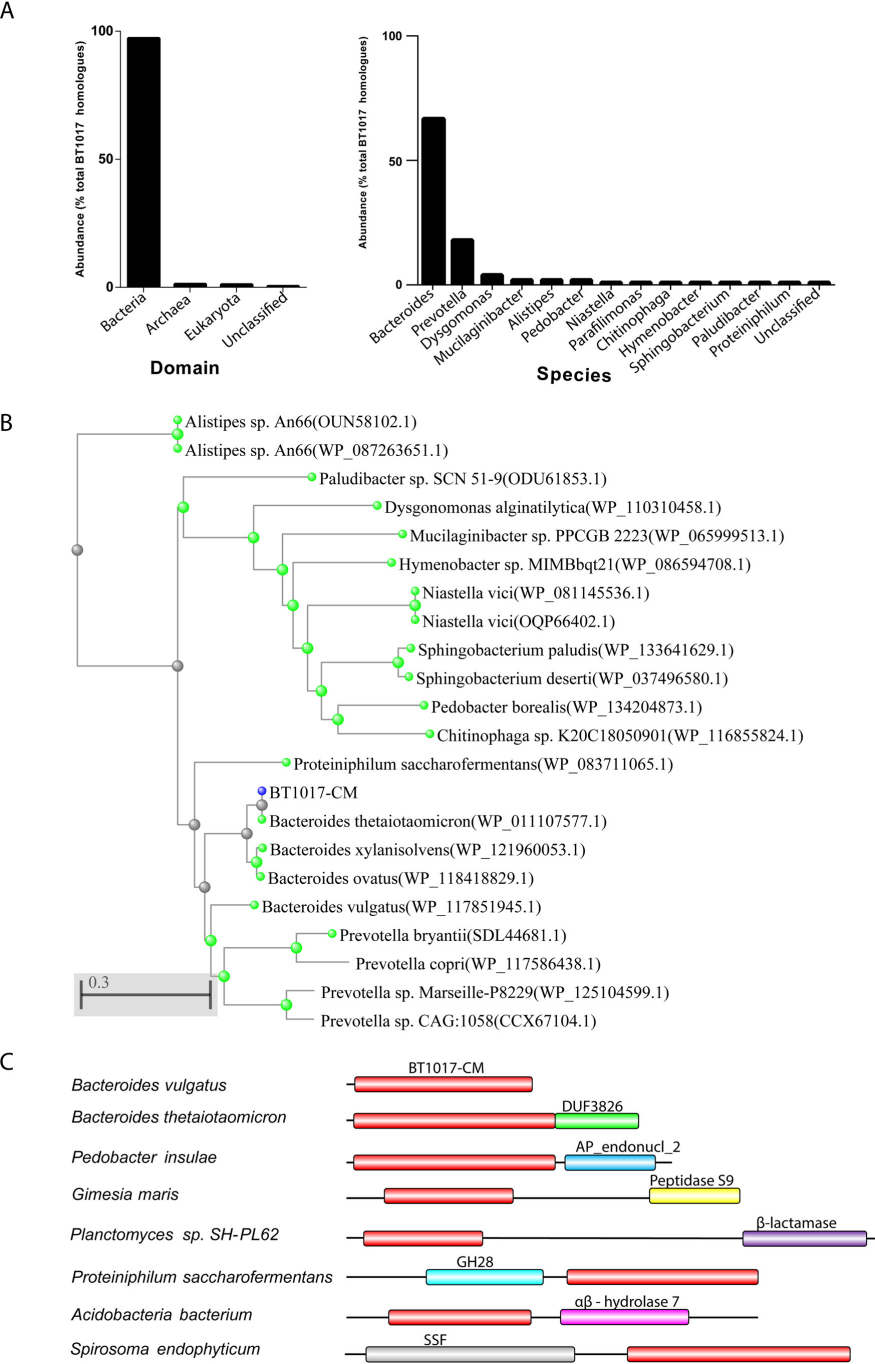
**Distribution of BT1017 homologs and family features.***A*, distribution of BT1017 homologs in various domains of life. *B*, phylogenetic analyses of BT1017-CM family members. *C*, typical architectures of BT1017 relatives in other domains of life.

An extensive HMM-(hidden Markov Model) based search using the HMMER web server (17) with the full-length BT1017 as query detected over 726 potential family members from all three domains of life (Fig. 7*A*). This family is dominated by bacterial sequences (97%), with 1.1% from Eukaryotes, 1.2% and Archaea. The remaining 0.4% were unclassified. The top hits (≥48%% identity to BT1017) were mostly distributed within reference genomes from members of the Bacteroidetes phylum with a majority of sequences (>90%) detected from members of the *Prevotella* and *Bacteroides* genera. A phylogenetic tree with selected sequences from various genera is shown in Fig. 7*B*. Data from a similar search with the BT1017-CM domain alone revealed varied domain architectures of BT1017-CM containing proteins. BT1017-CM domains for example were found associated with protein families such as GH28, peptidase S9, beta lactamase, solute symporter and xylose isomerase-like TIM barrel (Fig. 7*C*). This wide distribution and multiple domain associations suggest that BT1017-CM may be tailored to target substrates other than methylated pectins.

## Discussion

The metabolism of pectin by *B. thetaiotaomicron* has recently been elucidated, highlighting an array of CAZymes dedicated to the deconstruction of the RG-II domain (5, 18, 19). However, how RG-II structural variations due to methylation influence the enzymatic degradation of this polysaccharide is not fully understood.

BT1017 encoded by *B. thetaiotaomicron* is the founding member of a novel PME family shown to be essential to the metabolism of RG-II (5). Here, we show that a variant of apple RG-II, ΔBT1017oligoB, contains additional ester-linked methyl groups that are de-esterified by BT1017. Methyl esterification of backbone GalA and GlcA on side-chain A has previously been reported in RG-II from apple and *Arabidopsis thaliana* (5, 6). Whether one of the methyl groups detected in apple RG-II is attached to GlcA and/or to the backbone GalA of ΔBT1017oligoB remains to be determined. Our data clearly demonstrate that a single methyl esterase enzyme, BT1017, targets more than one methyl decoration in ΔBT1017oligoB. It is interesting to note that only a single acetyl esterase is required to remove the two acetate groups in RG-II side-chain B (5). This low sugar specificity among esterases is a common feature of this enzyme class. In contrast, each glycosidic bond in RG-II is hydrolyzed by a specific GH, reflecting tight specificity for these enzymes (5). The accumulation of this new and larger oligosaccharide (ΔBT1017oligoB) demonstrates that BT1017 has a greater impact on RG-II metabolism than previously thought.

The crystal structure of BT1017 was solved at 1.9 Å. To date, 18 carbohydrate esterase families have been identified with structural data reported for fourteen of them (9). The BT1017 α/β hydrolase fold is similar to many CEs that remove acetate groups from polysaccharides, exemplified by acetyl xylan esterases of families CE1 to CE7 (9). BT1017 was found to be structurally similar to acetyl xylan esterases despite showing methylesterase activity. The BT1017 fold differed from the only other known PME enzyme family, CE8, which displays β-helix fold. Our data showed that BT1017 is a canonical serine esterase deploying an Asp-His-Ser catalytic triad. This is in marked contrast to CE8 enzymes, which are aspartate esterases, therefore confirming the lack of an evolutionary link between the two PME families. The metal ion Zn^2+^ has been detected in a few esterase enzyme families including families CE4, CE11 and CE14 (9) where it has been demonstrated to play a role in catalysis. It will therefore be interesting to determine in future (*e.g.* through inhibition or mutational studies) whether Zn^2+^ plays a similar role in BT1017. The putative hydrophobic pocket that houses the methyl group and the oxyanion hole (composed of residues S282, G283 and H201) was identified in BT1017 by structural homology. However the uronic acid binding site could not be identified. To resolve this, the structure of a crystal complex of BT107-CM or its homologs with their methylated substrates will be required. BT1017-CM was also found to exhibit a generally low turnover (*k*_cat_ < 1 s^−1^) toward its substrate when compared with a most kinetically characterized pectin methylesterases and acetylesterases (20, 21, 22, 23, 24) characterized to date. The turnover was, however, in a similar range (*i.e. k*_cat_ < 1 s^−1^) as the characterized fungal glucoronyl esterases AaGE1 and PcGE1 from family CE15 (25). Methyl de-esterification may therefore be rate-limiting in the RG-II degradative process.

The BT1017-CM family showed a broad distribution across several gut species particularly the pectin-degrading genera *Bacteroides* and *Prevotella*, suggesting its importance for nutritional adaptation of this group of organisms in the human gut. It was also detected in organisms from diverse environments including soil, marine, and freshwater environments all habitats of diverse plant species, hinting that the PME class may be a critical adaptation for plant biomass–degrading microbes that are not restricted to the human gut.

## Conclusion

The current study sheds new light on the biochemical function and structure of the novel enzyme family represented by the BT1017 methyl esterase from *B. thetaiotaomicron*. The study not only enhances our general understanding of pectin metabolism by the HGM and related species from the environment but also shows how understanding degradative pathways in the HGM can yield new information on the structure of a target polysaccharide. This fundamental knowledge is required to inform and shape nutritional strategies that influence human health through the dietary manipulation of the HGM. Pectin methyl de-esterification is also part of physiological processes in plants, and this novel class of PMEs could have industrial applications for fruit ripening, pectin remodeling, and disease pathogenesis.

## Materials and methods

### Cloning and heterologous expression of BT1017 derivatives

The production of recombinant BT1017, BT1018, BT1021, BT1012, BT1001, BT0996, BT0997, BT0992, and BT1002 is as described by Ndeh *et al.* (5). For BT1017 constructs used to investigate the site of esterase activity in the protein, DNA sequences encoding various modules of BT1017 (BT1017-SP, BT1017-CM, and 1017-DUF3826) were amplified by PCR and cloned into pET-28a(+) vector (Novagen). All constructs were designed to contain the DNA sequence for a C-terminal polyhistidine tag (His_6_ tag). Recombinant constructs were se-quenced and used to transform *Escherichia coli* Tuner^TM^ (DE3) competent cells (Novagen). The cells were cultured in LB broth to exponential phase (*A*_600_ of 0.6), and protein expression was induced with 1 mm isopropyl β-d-thiogalactopyranoside. Induced cells were allowed to grow overnight at 16 °C and harvested the next day by centrifugation at 4000 × *g* for 10 min. The cells were resuspended in TALON® buffer (20 mm Tris-HCl, 150 mm NaCl, pH 8.0) (Clontech) and sonicated in ice, followed by high-speed centrifugation at 16,000 × *g* for 20 min. Recombinant proteins were purified from supernatants by immobilized metal-affinity chromatography using TALON® resins (Clontech). In brief, supernatants were applied to the resin bed equilibrated with TALON® buffer. The resin was washed with TALON® buffer, and proteins were eluted with increasing amounts of imidazole (10 and 100 mm) in TALON® buffer. Eluted fractions were analyzed using 12.5% SDS-PAGE, and pure fractions were buffer-exchanged into 20 mm NaH_2_PO_4_ buffer or other buffers of choice as indicated using a 10-kDa molecular mass filter concentrator (Amicon). Protein concentrations were estimated by absorbance at 280 nm using a NanoDrop^TM^ 2000/2000c spectrophotometer (Thermo Fisher Scientific) and respective molar extinction coefficients for each recombinant protein (BT1017-SP = 77,045 m^−1^ cm^−1^, BT1017-CM = 44,155 m^−1^ cm^−1^, BT1017-DUF3826 = 31,400 m^−1^ cm^−1^, BT1018 = 33,055 m^−1^ cm^−1^, BT1021 = 62,340 m^−1^ cm^−1^, BT1012 = 133,160 m^−1^ cm^−1^, BT1001 = 190,040 m^−1^ cm^−1^, BT0996 = 340,310 m^−1^ cm^−1^, BT0997 = 166,425 m^−1^ cm^−1^, BT0992 = 182,590 m^−1^ cm^−1^, and BT1002 = 128,855 m^−1^ cm^−1^).

### Enzyme kinetics

Kinetic assays were performed using a coupled spectrophotometric enzyme assay that measures the amount of released methanol as described by Grsic-Rausch and Rausch (11). A standard reaction mixture contained varying amounts of substrate and enzyme (1.7 μm) in a final volume of 500 µl. The concentration range was 0.09–3.47 mm for ΔBT1017oligoA, 0.046–1.38 mm for ΔBT1017oligoB, 0.0032–3.2 mm for GalMe, 0.028–2.8 mm for GlcAMe, 0.006–0.17 mm for apple pectin, and 0.006–0.111 mm for apple RG-II. Methanol release was monitored using an Ultrospec 4000, UV-visible spectrophotometer (Pharmacia Biotech). All tests were performed in triplicate. To assess the impact of temperature and pH on BT1017 activity, the reactions were performed at different temperatures (20, 25, 30, 35, 40, 45, and 50 °C) and pH values (pH 6.5, 7, 7.5, 8. 8.5, and 9.0). The buffers used were 50 mm NaH_2_PO_4_ for the pH range of 6.5–7.5 and 50 mm Bis-Tris-propane for the pH range of 8.0–9.0.

### TLC

Enzymatic reactions were stopped by heating for 3 min at 98 °C and centrifuged for 1 min at 17,000 × *g*. For each reaction, 4 µl (2 µl × 2) was spotted onto a silica gel 60 TLC plate (Merck), and sugars were resolved in running buffer containing butanol/acetic acid/water (2:1:1, v/v/v). At the end of the run, the plates were dried and treated with orcinol sulfuric acid reagent (sulfuric acid/ethanol/water in the ratio 3:70:20 v/v/v and 0.5% orcinol). Sugar bands were detected by gently heating the aluminum support of the plates over a Bunsen flame.

### HPLC

Samples initially heated and centrifuged as described above for TLC were injected into a Dionex CARBOPAC^TM^ HPLC system fitted with a Dionex^TM^ CarboPac^TM^ PA1 anion-exchange column (Dionex). A typical HPLC run consisted of two elution phases: an isocratic phase where 100 mm NaOH is pumped (flow rate of 1.0 ml/min) through the column for 30 min followed by another 30 min of a gradient phase during which an increasing amount of 500 mm NaOAc (0–100%) was pumped through the column (flow rate of 1.0 ml/min). Eluates were monitored by pulsed amperometric detection with a fitted EC detector. HPLC data were analyzed using Chromeleon^TM^ chromatography software (version 6.8, Dionex) and GraphPad Prism (version 7.0, Prism).

### Digestion of ΔBT1017oligoA and B with ΔBT1017oligoA-degrading enzymes

Each substrate (4.8 mg/ml) was treated for 4 h with a mixture of A5 or ΔBT1017oligoA-degrading enzymes **(**BT1017, BT1018, BT1021, BT1012, and BT1001). The concentration of each enzyme in the mixture was 0.4 μm. The reactions were stopped by boiling for 5 min. Volumes corresponding to 9.6 and 24 µg of the digested substrates were analyzed by TLC and HPLC, respectively.

### Digestion of ΔBT1017oligoB with BT1017 and BT0996

The ΔBT1017oligoB substrate (4.3 mg/ml) was treated overnight with 0.7 μm each of BT1017 and BT0996. The reactions were stopped by boiling for 5 min, and 8.6 µg of digested sample was analyzed by HPLC as described above.

### Isothermal titration calorimetry

Recombinant proteins were tested for binding activity toward RG-II by isothermal titration calorimetry as described previously (26). Titrations were carried out in 50 mm HEPES buffer, pH 7.5, at 25 °C. The concentration of apple RG-II in the syringe was 10 mg/ml, and the concentration of each protein was 100 μm.

### Site-directed mutagenesis of BT1017

BT1017 active site mutants alanine mutants S282A, D352A, and H388A were generated using a QuikChange^TM^ site-directed mutagenesis kit (Stratagene) with high fidelity KOD DNA polymerase (Novagen). The mutants were confirmed by sequencing DNA purified from selected clones at Eurofins Genomics.

### Growth of B. thetaiotaomicron WT and mutant strains and HPLC analyses of growth supernatants

*B. thetaiotaomicron* strains were cultured at 37 °C in an anaerobic cabinet (WhitleyA35 workstation; Don Whitley). The cells were initially prepared in 5 ml of tryptone–yeast–glucose culture medium overnight before growth in minimal medium containing various substrates as previously reported (27). For the preparation of ΔBT1017oligoA and ΔBT1017oligoB substrates, WT and mutant strains were cultured in 50 ml of minimal medium containing 4% CEAJ powder (5) for 48 h (at the stationary phase, *A*_600 nm_ of ∼1.0) before purification. For growth samples destined for HPLC analyses, 1% of the substrate (in 0.2 ml of minimal medium) was used. Growth substrates included ΔBT1017oligoB, purified apple RG-II, and CEAJ powder (5). Cultures at stationary phase were centrifuged, and supernatants were boiled at 98 °C for 3 min. The samples were allowed to cool and centrifuged at 17,000 × *g*, and 10 µl of each sample was analyzed by HPLC.

### Purification of ΔBT1017oligoA and B

Spent media at the stationary phase (*A*_600 nm_ of ∼1.0) of *B. thetaiotaomicron* ΔBT1017 grown on CEAJ (50 ml) were centrifuged twice at 4000 × *g* for 6 min. The presence of sugars in the supernatants was confirmed by TLC as described above. Supernatants were filtered through a 0.2-μm syringe cap filter (PALL Life Sciences) and concentrated by freeze-drying using a CHRIST Gefriertrocknung ALPHA 1-2 freeze-dryer (Helmholtz-Zentrum Berlin) at −50°C to reduce the sample volume (to 20 ml). Sugars in supernatant were separated on a Bio-Gel P2 (Bio-Rad) size-exclusion system equilibrated in 5 mm acetic acid at a flow rate 0.6 ml min^−1^. Fractions (8 ml) were collected and analyzed by TLC. Fractions of interest were pooled and concentrated by freeze-drying and stored at room temperature until use.

### MS and NMR

After Bio-Gel®-P2 gel filtration and freeze-drying, digested or undigested ΔBT1017oligoB was dissolved in distilled water and analyzed by infusion electrospray ionization–MS. The analysis was performed as per Ndeh *et al.* (5) with the following modifications. Positive ion mode analysis was performed on the desalted oligosaccharides (in 10 mm ammonium acetate, pH 7.0) by dilution 1:10 (v/v) into 10 mm ammonium formate, pH 3.0, containing 20% acetonitrile. This was followed by infusion electrospray ionization–MS using an LTQ-FT mass spectrometer (Thermo) with the acquisition and data analysis settings described previously (5). For NMR, ΔBT1017oligoB (∼10 mg) was prepared by dissolving it in D_2_O (0.6 ml), and brief centrifugation was required to clear the solution from impurities. The spectra were recorded using Bruker Avance NEO 600 MHz NMR spectrometer equipped with TCI CryoProbe. The data were collected in D_2_O at 25 °C using acetone (δ_H_ 2.17, δ_C_ 30.9) as an internal reference and processed using Mnova software.

### Expression of selenomethionine substituted BT1017-CM

The recombinant plasmid pET-28-1017-CM was transformed into the Met-auxotrophic *E. coli* B834 and positive clones were selected by growth on kanamycin medium. A streak of B834 colonies were inoculated into 5 ml of LB containing 10 µg/ml kanamycin and grown at 37 °C overnight. The culture was then inoculated into 100 ml of LB supplemented with 10 µg/ml kanamycin and grown at 37 °C until *A*_600 nm_ = 0.2–0.4. The cells were harvested and resuspended with 100 ml of H_2_O. This step was repeated three times to remove remaining LB medium. The final resuspended cell pellet was inoculated into 1 liter of SelenoMet Medium Base^TM^, which contained 50 ml of SelenoMet^TM^ nutrient mix and 4 ml of selenomethionine solution (10 mg/ml) (Molecular Dimensions). The expression of selenomethionine proteins was performed as described for the native form. Selenomethionine proteins were purified with TALON® resin and then further purified with gel filtration column (Hiload^TM^ 16/600, Superdex^TM^ 200 pg). Selenomethionine proteins were concentrated and buffer-exchanged into 10 mm HEPES (pH 7.5) containing 150 mm NaCl to a final concentration of 10 mg/ml for crystal screening.

### Structure determination of BT1017

After immobilized metal-affinity chromatography, purification and gel filtration samples of interest were collected, pooled, and buffer-exchanged by centrifugation using a 10-kDa molecular mass filter concentrator (Amicon). The final sample contained 200 μm of BT1017-CM in 10 mm HEPES and 150 mm NaCl. The protein was crystallized at 20 °C using the sitting-drop vapor-diffusion method using a Mosquito dispensing robot (SPT Labtech). The protein:reservoir ratios were 1:1 and 2:1 with final drop volumes of 200 and 300 nl, respectively. The crystallization conditions were Morpheus (Molecular Dimensions) conditions D2 (120 mm alcohols, 100 mm buffer system 1, pH 6.5, 30% ethylene glycol/PEG 8000). Diffraction data were collected at Synchrotron Beamline I24 of Diamond Light Source (Didcot, UK) at a temperature of 100 K. The data were processed and integrated using iMOSFLM and scaled using Aimless (19, 28). Space group determination was tested using POINTLESS and confirmed during refinement (29). The crystal structure of BT1017 was solved using single-wavelength anomalous dispersion based on the selenomethionine sites. The sites and phases were determined using SHELXC/D/E pipeline with HKL2MAP (30). The model was built with Buccaneer in CCP4 (31). The model was completed using iterative cycles of refinement with refmac5 and model-building using COOT (13, 32). The model was refined against the anomalous data, and selenomethionine residues were built in place of methionines. The *R*_free_ set of reflections was 5% of the unique reflections randomly selected. The model was validated using Coot and MolProbity (12, 13).

### Inductively coupled plasma–MS

BT1017-CM was dialyzed overnight in buffer containing 10 mm Tris, pH 8.0, and 50 mm NaCl overnight, freeze-dried, and digested with ultrapure nitric acid and hydrogen peroxide. The digest was then diluted in Rhodium internal standard and Milli-Q water. The elemental content of the sample was determined using a thermon TQ triple quad spectrometer with the following operating conditions: cooling flow rate, 14.0 liters/min; auxillary gas flow rate, 0.8 liters/min; sampling depth, 5 mm; additional gas flow, 75%; spray chamber, 2.7 °C; nebulizer flow rate, 1.144//min; pump speed, 15 rpm; and RF power, 15550 W.

### In silico and phylogenetic analyses

The HMMER web server (14) was queried with BT1017-CM and full-length BT1017 to recover matching sequences from all three domains of life. BLASTP analyses (NCBI database, www.ncbi.nlm.nih.gov) were performed using BT1017-CM as query and selected proteins from major genera represented were used to generate a distance tree. The latter was based the neighborhood joining method (maximum sequence difference, 0.85; distance, Grishin).

## Data availability

All data are contained within the article. Structural data for BT1017-CM are also available in the Protein Data bank under accession number 6GOC.
