# Supplementary material for: Ascertaining the biochemical function of an essential pectin methylesterase in the gut microbe Bacteroides thetaiotaomicron
Source: J Biol Chem. 2021 Jan 13;295(52):18625–37. doi: 10.1074/jbc.RA120.014974 (PMC7939467; doi:10.1074/jbc.RA120.014974)
Supplement: Supplementary file 1 [file mmc1.zip › 161769_2_supp_613881_qsbcs6.pdf]

Analytes	Isotope measured	Amount (µmoles)
Mg	25Mg (S-SQ-KED)	0.176
P	31P | 31P.16O (S-TQ-O2)	14.736
S	32S | 32S.16O (S-TQ-O2)	145.589
Ca	44Ca (S-SQ-KED)	1.929
V	51V | 51V.16O (S-TQ-O2)	-0.001
Cr	52Cr | 52Cr.16O (S-TQ-O2)	0.006
Mn	55Mn | 55Mn.16O (S-TQ-O2)	0.024
Fe	56Fe | 56Fe.16O (S-TQ-O2)	0.216
Co	59Co (S-SQ-KED)	1.141
Ni	60Ni | 60Ni.16O (S-TQ-O2)	0.562
Zn	64Zn (S-SQ-KED)	10.870
Cu	65Cu | 65Cu.16O (S-TQ-O2)	0.106
Mo	92Mo (S-SQ-KED)	-0.152

Supplemental table 1: ICP-MS analyses of BT107-CM metal ion content
