# Supplementary material for: Ascertaining the biochemical function of an essential pectin methylesterase in the gut microbe Bacteroides thetaiotaomicron
Source: J Biol Chem. 2021 Jan 13;295(52):18625–37. doi: 10.1074/jbc.RA120.014974 (PMC7939467; doi:10.1074/jbc.RA120.014974)
Supplement: Supplementary file 1 [file mmc1.zip › 161769_2_supp_613882_q5bc56.pdf]

#	Scoring help			RMSD	Nalign	Ng	%seq	Query	Target (PDB entry)			Title
	Q	P	Z					%sse	Match	%sse	Nres	
1	0.25	2	11	1.85	245	18	20	44	3nuz:A	48	388	CRYSTAL STRUCTURE OF A PUTATIVE ACETYL XYLAN ESTERASE (BF1801) FROM BACTEROIDES FRAGILIS NCTC 9343 AT 2.30 A RESOLUTION
2	0.25	3.1	10.8	1.85	244	20	19	44	3nuz:B	48	389	CRYSTAL STRUCTURE OF A PUTATIVE ACETYL XYLAN ESTERASE (BF1801) FROM BACTEROIDES FRAGILIS NCTC 9343 AT 2.30 A RESOLUTION
3	0.25	2.6	11.3	1.86	244	19	20	44	3nuz:D	52	389	CRYSTAL STRUCTURE OF A PUTATIVE ACETYL XYLAN ESTERASE (BF1801) FROM BACTEROIDES FRAGILIS NCTC 9343 AT 2.30 A RESOLUTION
4	0.25	2.9	10.7	1.84	242	20	20	44	3nuz:F	48	386	CRYSTAL STRUCTURE OF A PUTATIVE ACETYL XYLAN ESTERASE (BF1801) FROM BACTEROIDES FRAGILIS NCTC 9343 AT 2.30 A RESOLUTION
5	0.24	4.5	11	1.98	247	18	20	44	3g8y:A	48	391	CRYSTAL STRUCTURE OF A PUTATIVE HYDROLASE (BVU_4111) FROM BACTEROIDES VULGATUS ATCC 8482 AT 1.90 A RESOLUTION
6	0.24	3	11.1	1.84	239	18	20	44	3nuz:E	52	385	CRYSTAL STRUCTURE OF A PUTATIVE ACETYL XYLAN ESTERASE (BF1801) FROM BACTEROIDES FRAGILIS NCTC 9343 AT 2.30 A RESOLUTION
7	0.24	1.3	11	1.86	238	17	19	41	3nuz:C	48	388	CRYSTAL STRUCTURE OF A PUTATIVE ACETYL XYLAN ESTERASE (BF1801) FROM BACTEROIDES FRAGILIS NCTC 9343 AT 2.30 A RESOLUTION
8	0.19	4.2	9.2	2.45	235	22	18	41	6gu8:A	52	392	GLUCURONOYL ESTERASE FROM SOLIBACTER USITATUS
9	0.19	3.4	9.3	2.45	234	21	17	41	6gry:A	52	392	GLUCURONOYL ESTERASE FROM SOLIBACTER USITATUS.
10	0.17	1.6	8.1	2.46	190	20	11	41	1tht:A	59	294	STRUCTURE OF A MYRISTOYL-ACP-SPECIFIC THIOESTERASE FROM VIBRIO HARVEYI
11	0.16	1.6	8.1	2.45	189	21	11	41	1tht:B	59	293	STRUCTURE OF A MYRISTOYL-ACP-SPECIFIC THIOESTERASE FROM VIBRIO HARVEYI
12	0.16	3.5	9.7	2.45	195	12	11	41	5hdp:D	57	316	HYDROLASE STNA MUTANT - S185A
13	0.16	0.6	8.9	2.55	184	15	14	31	3e3a:B	45	275	THE STRUCTURE OF RV0554 FROM MYCOBACTERIUM TUBERCULOSIS
14	0.16	2.6	8.2	2.78	202	15	10	41	4zxf:A	65	308	CRYSTAL STRUCTURE OF A SOLUBLE VARIANT OF MONOGLYCERIDE LIPASE FROM SACCHAROMYCES CEREVISIAE IN COMPLEX WITH A SUBSTRATE ANALOG
15	0.16	2.8	10.1	2.43	193	13	10	41	5hdp:G	57	316	HYDROLASE STNA MUTANT - S185A
16	0.16	2.8	9.5	2.44	190	13	19	41	5xb6:H	59	306	CRYSTAL STRUCTURE OF YCJY FROM E. COLI
17	0.16	1.5	8.3	2.74	200	15	10	41	4zxf:B	59	307	CRYSTAL STRUCTURE OF A SOLUBLE VARIANT OF MONOGLYCERIDE LIPASE FROM SACCHAROMYCES CEREVISIAE IN COMPLEX WITH A SUBSTRATE ANALOG
18	0.16	1.2	8.7	2.45	180	13	14	34	3e3a:A	50	274	THE STRUCTURE OF RV0554 FROM MYCOBACTERIUM TUBERCULOSIS
19	0.16	2.7	9.7	2.42	191	12	11	41	5hdp:F	59	314	HYDROLASE STNA MUTANT - S185A
20	0.16	3	9.7	2.51	191	12	19	41	5xb6:F	59	306	CRYSTAL STRUCTURE OF YCJY FROM E. COLI

Supplemental table 2: Details of structural homologues of BT1017-CM. Homologues were searched using the online resource PDBeFold (27) with the structure of BT1017-CM as query.
