# Supplementary material for: Ascertaining the biochemical function of an essential pectin methylesterase in the gut microbe Bacteroides thetaiotaomicron
Source: J Biol Chem. 2021 Jan 13;295(52):18625–37. doi: 10.1074/jbc.RA120.014974 (PMC7939467; doi:10.1074/jbc.RA120.014974)
Supplement: Supplementary file 1 [file mmc1.zip › 161769_2_supp_613883_q3bc36.pdf]

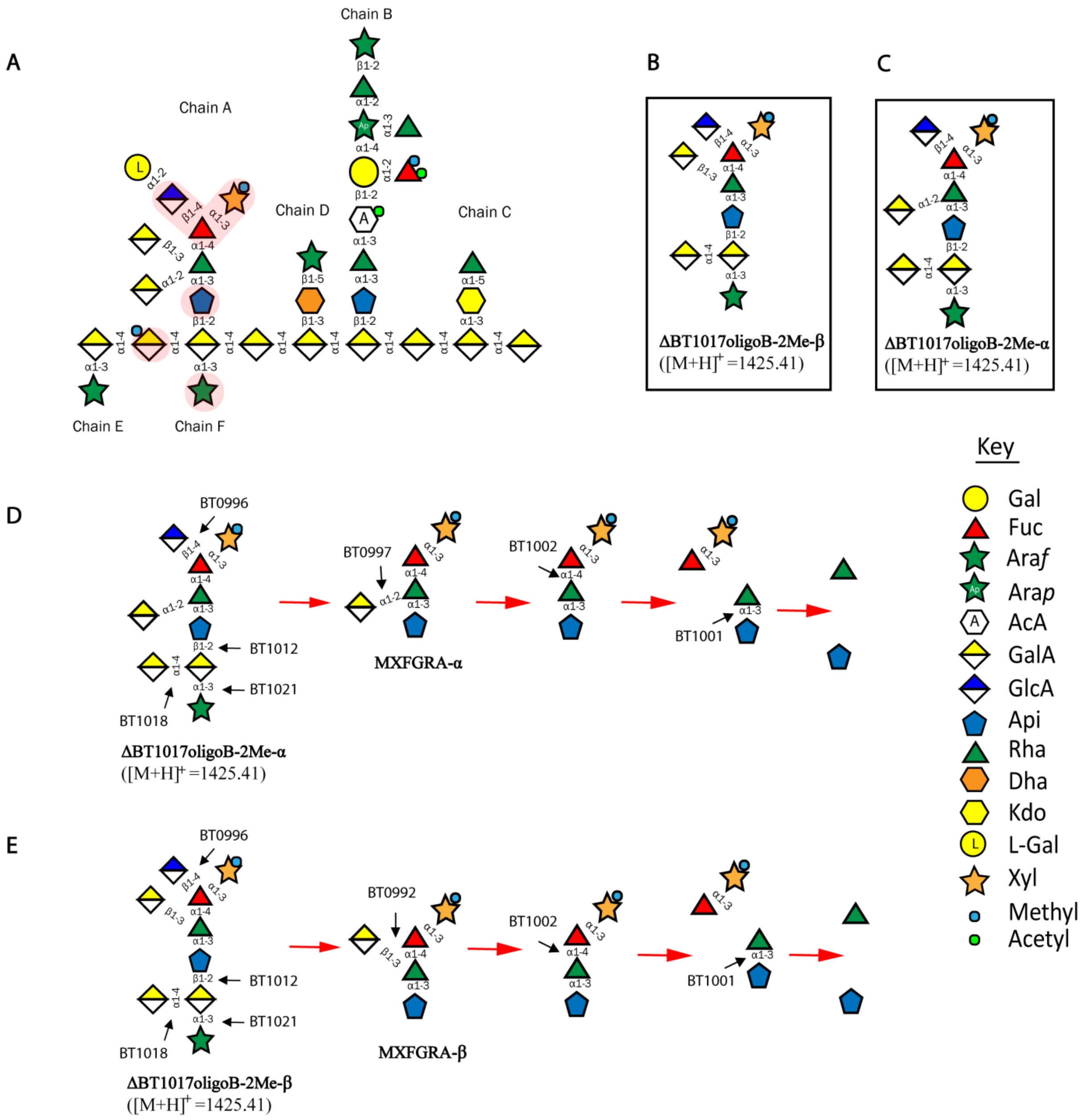

Supplemental figure 1: Uncovering the identity of ΔBT1017oligoB. A) Structure of RG-II showing structural components detected in ΔBT1017oligoB (pink highlights). **B/C)** The structures of ΔBT1017oligoB-α and ΔBT1017oligoB-β. **D/E)** Series of steps and enzymes required for the complete degradation of ΔBT1017oligoB-2Me-α and ΔBT1017oligoB-2Me-β as reported by Ndeh *et al.*, 2017 (5)
