# Supplementary material for: Ascertaining the biochemical function of an essential pectin methylesterase in the gut microbe Bacteroides thetaiotaomicron
Source: J Biol Chem. 2021 Jan 13;295(52):18625–37. doi: 10.1074/jbc.RA120.014974 (PMC7939467; doi:10.1074/jbc.RA120.014974)
Supplement: Supplementary file 1 [file mmc1.zip › 161769_2_supp_613884_q6bc66.pdf]

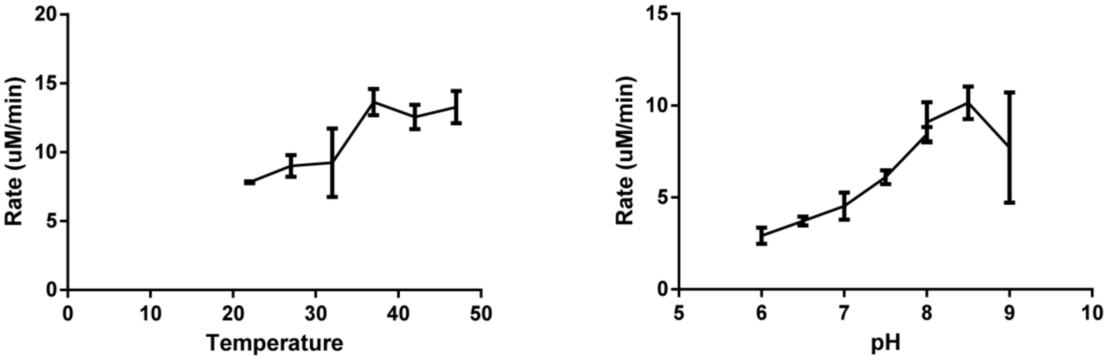

Supplemental figure 2: Effects of temperature and pH on BT1017 activity. A) Temperature dependence. **B)** pH dependence. The buffers used included 50 mM NaH_2PO_4 in the range between pH 6.5 and 7.5 and 50 mM BIS-TRIS Propane between pH 8.0 and 9.0
