# Supplementary material for: Ascertaining the biochemical function of an essential pectin methylesterase in the gut microbe Bacteroides thetaiotaomicron
Source: J Biol Chem. 2021 Jan 13;295(52):18625–37. doi: 10.1074/jbc.RA120.014974 (PMC7939467; doi:10.1074/jbc.RA120.014974)
Supplement: Supplementary file 1 [file mmc1.zip › 161769_2_supp_613885_q5bc56.pdf]

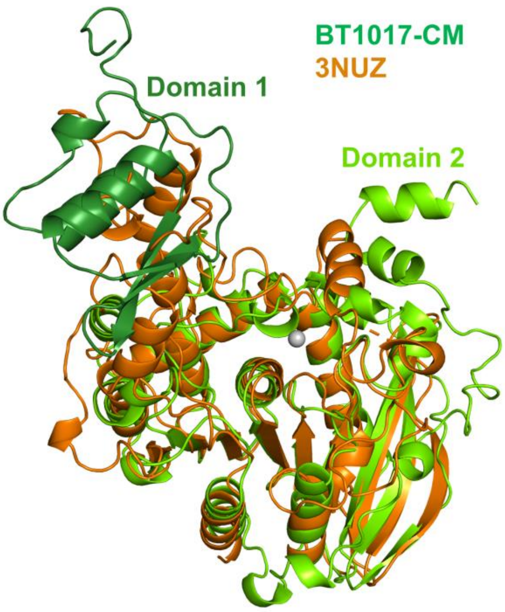

Supplemental figure 3: Structural superposition of BT1017-CM and a xylan acetyl esterase BF1801 (3NUZ) from *B. fragilis*. BT1017-CM is presented in green and BF1801 in orange. The superposition shows significant alignment of sections of the central a/b hydrolase fold within BT1017-CM and BF1801. Many loop structures surrounding the substrate binding site are however out of sync and BF1801 lacks a metal ion (grey ball in the binding site represents metal ion for BT1017-CM). An N-terminal loop and to two α-helices in BF1801 occupies the crystallographic position of the domain 1 (dark green) from BT1017-CM.
