# Supplementary material for: Ascertaining the biochemical function of an essential pectin methylesterase in the gut microbe Bacteroides thetaiotaomicron
Source: J Biol Chem. 2021 Jan 13;295(52):18625–37. doi: 10.1074/jbc.RA120.014974 (PMC7939467; doi:10.1074/jbc.RA120.014974)
Supplement: Supplementary file 1 [file mmc1.zip › 161769_2_supp_613886_qcbcc6.pdf]

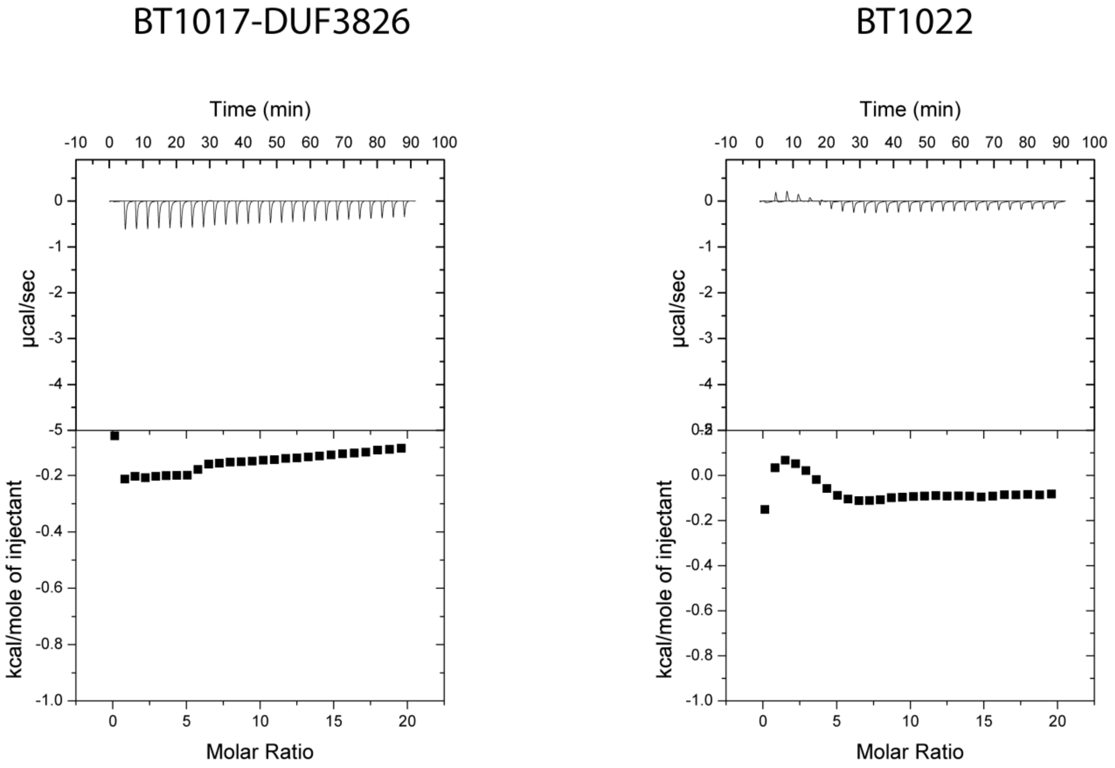

Supplemental figure 4: Representative isothermal titration calorimetry (ITC) plots for BT1017-DUF3826 and BT1022's interaction with apple RG-II. The top halves of each dataset show raw ITC heats while the bottom halves represent corresponding changes in released energy per mole of injectant.
