# Supplementary figures and images for: Ascertaining the biochemical function of an essential pectin methylesterase in the gut microbe Bacteroides thetaiotaomicron
Source: J Biol Chem. 2021 Jan 13;295(52):18625–37. doi: 10.1074/jbc.RA120.014974 (PMC7939467; doi:10.1074/jbc.RA120.014974)

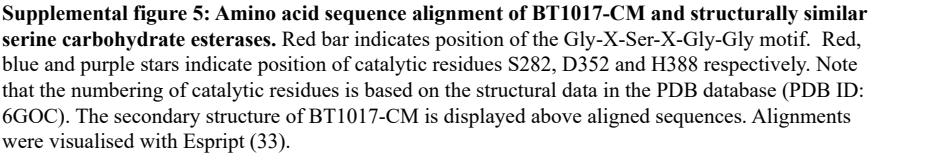

Supplement: Supplementary file 1 [file mmc1.zip › 161769_2_supp_613902_q3bh43.pdf]
